# Supplementary material for: Pericardial Fluid Accumulates microRNAs That Regulate Heart Fibrosis after Myocardial Infarction
Source: Int J Mol Sci. 2024 Jul 30;25(15):8329. doi: 10.3390/ijms25158329 (PMC11313565; doi:10.3390/ijms25158329)
Supplement: Supplementary file 1 [file ijms-25-08329-s001.zip › Supplementary Materials.pdf]

Supplementary Materials

Supplementary Table S1

Primer sequences used in qRT-PCR for human and mouse genes.

Gene	Primer Sequence (5′ to 3′)
ACTA2	RV: GTGGTTTCATGGATGCCAGC<br>FW: GGCAAGTGATCACCATCGGA
COL1A1	RV: CAGATCACGTCATCGCACAAC<br>FW: GAGGGCCAAAGACGAAGACATC
CCN2	RV: CCGTCGGTACATACTCCACAGA<br>FW: CTTGCGAAGCTGACCTGCAAGA
GAPDH	RV: GGACTCCCCAGCAGTG<br>FW: CCTCCACCTTTGACGCT

Supplementary Table S2

miRs and respective normalized counts (counts/million, Chimera software) [66] identified in all samples subjected to RNA sequencing

miRNA	CTRL	CTRL	CTRL	CTRL	CTRL	CTRL	CTRL	CTRL	NSTEMI	NSTEMI	NSTEMI	NSTEMI	NSTEMI	NSTEMI	NSTEMI	NSTEMI	STEMI	STEMI	STEMI	STEMI	STEMI	STEMI
**hsa-let-7a-2-3p**	421198	354125	336063	170021	340134	474327	172806	296210	361548	298111	381473	178015	478965	404631	234622	611384	438950	653309	207619	477456	1944613	505521
**hsa-let-7a-3p**	168271	187811	128353	164647	156387	129740	118672	177248	161824	126147	149087	166855	91892	93876	90381	107920	233484	86105	143526	107212	71326	125612
**hsa-let-7a-5p**	133662	56936	67996	55053	46100	190482	55841	1964	38579	198978	46329	15090	35357	551631	1008871	14525	12501	17406	14535	74470	217638	40938
**hsa-let-7b-3p**	97228	81497	64688	111496	99512	70191	87052	73511	70186	79958	73204	121276	72704	75532	38212	55505	89070	65158	72735	74974	32867	82532
**hsa-let-7b-5p**	65680	47559	76352	55347	69378	69014	62637	94532	109008	76170	132856	100757	69597	68787	69691	84084	100281	89890	74206	78348	25728	85610
**hsa-let-7c-3p**	63132	79238	42108	54489	41956	28626	34551	26650	36260	29201	63672	47090	33157	30843	28055	23732	53616	36795	66173	25136	25443	27595
**hsa-let-7c-5p**	61044	139459	141354	123618	140726	109999	150603	165263	89218	114955	89132	127428	108448	119303	82215	74996	128581	112855	209217	154387	89555	87894
**hsa-let-7d-3p**	52071	84539	102889	55592	83020	47163	97471	75634	84493	70752	105830	73116	55160	62541	68674	65325	91809	121925	56463	80675	12937	64235
**hsa-let-7d-5p**	50611	47158	38338	55599	59656	47265	29411	39420	48654	41007	54448	45972	60846	53375	20731	43153	56068	29312	45773	38578	94048	44147
**hsa-let-7e-3p**	50230	21687	17120	41071	16214	67451	13430	34473	10683	40528	8893	23770	36707	71194	32701	26692	26172	9956	42585	21155	61870	29214
**hsa-let-7e-5p**	48704	23423	31499	71903	32707	72358	28013	91889	29221	62415	34801	43090	59863	40329	56469	62884	39131	39168	68040	27494	39496	57606
**hsa-let-7f-1-3p**	48155	14468	26970	12946	21795	15706	14021	24245	30379	20955	40041	17882	18285	16546	14138	28209	43463	28529	22246	27738	41723	33579
**hsa-let-7f-2-3p**	38681	34139	49458	59071	62255	52337	53561	32124	63931	73887	69400	83847	45209	57566	30968	46624	55828	27375	47808	47179	58996	40237
**hsa-let-7f-5p**	34881	34943	24700	48406	30734	24213	30186	61720	32701	26551	23580	27339	24357	20117	42606	31069	29458	43281	42735	16903	17133	31139
**hsa-let-7g-5p**	34143	53905	44531	50350	53000	32748	41344	43057	57031	46998	51173	54598	60091	32573	23187	38519	68734	35550	34071	39984	37025	42315
**hsa-let-7i-3p**	27446	24201	48015	17131	53850	21873	27779	17949	68730	45376	70671	41494	33183	56902	31390	38239	83268	36229	25128	47892	16280	43138
**hsa-let-7i-5p**	26298	29077	22649	44884	31503	19465	25476	51594	33634	24248	27560	28262	21720	19525	29058	28025	30204	37585	38968	17004	13185	31657
**hsa-mir-100-3p**	26172	33677	21119	46892	25663	18221	17352	48314	38509	24714	23746	31087	23482	13415	42770	28229	23834	36486	34526	11826	11368	27953
**hsa-mir-100-5p**	25696	14067	26182	14545	20854	22322	16065	27715	36166	28166	43112	20999	21604	22446	24631	33978	30333	36559	26328	28846	29132	25731
**hsa-mir-101-3p**	23226	31341	27777	53850	24532	50724	35229	44814	12937	40957	8836	38246	41217	23351	27201	17535	21489	25468	40221	20881	16863	17959
**hsa-mir-101-5p**	22747	30424	33816	15343	18594	26902	35178	12795	23473	26995	21295	31509	16599	27111	18775	16143	21554	23005	19469	24652	24373	11289

**hsa-mir-103a-2-5p**	18083	22546	19687	13967	20424	18304	12199	6114	12778	17684	12142	15525	12195	16739	6657	8865	17139	8768	14784	11431	9645	10977
**hsa-mir-103a-3p**	17227	20250	16070	19968	14172	20800	18079	27559	18543	13853	18079	14786	16892	14637	26767	18781	16475	23504	23269	13470	11859	19173
**hsa-mir-105-5p**	16844	12241	11128	3093	15248	12472	6066	14699	15952	5412	15038	8025	52956	28177	13450	21310	21801	13558	10450	19568	52104	25612
**hsa-mir-106a-3p**	16469	19406	14289	39603	23186	14957	10672	22276	13250	19683	11577	21976	16842	14037	8642	13615	20776	11651	19493	12252	8996	16658
**hsa-mir-106a-5p**	15229	20093	11200	81256	9810	25618	11663	56930	4812	22893	1847	19724	31396	9774	8101	17335	8773	10147	28817	7841	5750	13002
**hsa-mir-106b-3p**	14951	5349	3173	16134	3689	13344	1301	16117	2072	10263	1879	6203	19085	6572	3756	10687	8990	1857	21929	3670	17049	10444
**hsa-mir-106b-5p**	14645	16323	16034	9321	18564	27210	16299	9691	19820	19784	21072	9327	18125	38840	88609	13419	8563	14934	11657	15714	24697	12780
**hsa-mir-107**	14484	17706	15246	12368	14801	15812	20372	22419	15957	15906	19527	12922	16632	21205	52903	14178	15926	25340	13754	18196	25002	12654
**hsa-mir-10a-3p**	13116	19496	10318	22004	15704	9572	6134	13720	9450	12412	7254	13858	15609	7528	3725	11299	14768	6125	13757	10556	8439	11476
**hsa-mir-10a-5p**	12374	28433	24990	15634	26299	24810	26531	26540	35424	24824	16554	19853	45528	17079	14789	39527	16728	28526	22428	25446	24055	25681
**hsa-mir-10b-3p**	10980	12881	13325	6632	13747	6799	9285	7799	14430	10416	15203	11801	11170	8798	7780	9963	12263	11024	7824	9407	10401	9567
**hsa-mir-10b-5p**	9600	14897	15861	24093	14613	10235	34178	28450	14114	13866	12036	13494	12523	10100	11596	12283	15446	22552	20680	13832	5837	13581
**hsa-mir-1178-3p**	9019	4392	5469	12020	7757	9014	4818	5384	5642	8313	5870	7800	7834	12994	7241	9257	11540	3209	5215	7598	22396	8671
**hsa-mir-1178-5p**	8114	10316	11488	17808	10869	11650	10084	10257	9300	9784	11208	12449	13193	10112	2608	10533	12622	6505	11005	12582	12970	10969
**hsa-mir-1179**	8077	14177	13355	24146	12528	8726	14941	16041	11897	12373	7009	11785	11332	5436	10058	10312	8259	14020	17520	7677	4090	10297
**hsa-mir-1180-3p**	7750	4854	11603	3728	7945	4159	11551	2330	7733	4420	8417	4690	12738	18690	26364	4663	8290	13186	5072	12262	18126	9995
**hsa-mir-1180-5p**	6574	11542	6697	21578	6340	12991	5700	15740	5442	16180	2822	15423	17126	5423	7910	9880	5412	5156	15291	3908	2690	9023
**hsa-mir-1181**	6270	2005	2628	1539	2056	727	2107	1377	1247	1524	4875	1978	1702	3838	3699	1182	2074	960	954	1505	6225	1743
**hsa-mir-1183**	6264	3471	4189	9598	5282	8271	6379	9245	6086	3475	4498	5120	5058	4403	6539	6561	5979	6964	8709	4109	4397	7633
**hsa-mir-1184**	6127	8205	6695	17642	8894	5005	7250	15723	9345	7391	7504	8619	6813	4779	3560	7904	8343	10213	11940	5145	3680	8098
**hsa-mir-1185-1-3p**	5792	3668	1603	4582	1825	1348	1498	4011	1405	1381	3306	1556	588	2025	2701	1101	2279	5169	1623	1247	1070	1892

**hsa-mir-1185-2-3p**	5102	3784	9076	3614	5887	4279	7480	6060	6620	4324	3993	5284	4900	3219	2121	4033	5704	4667	6162	3827	1195	6414
**hsa-mir-1185-5p**	4919	14209	17659	12616	18919	16119	14450	15535	14515	17744	15845	17785	18012	20005	17416	13715	15036	14796	13415	16430	8626	12158
**hsa-mir-1193**	4853	5185	10674	5536	9668	5975	8542	6474	13069	8903	10583	7860	6008	8328	16622	7418	8379	11918	4764	6720	1694	9164
**hsa-mir-1197**	4839	4308	6372	4565	5005	8858	8919	4974	6596	8059	8161	4373	4221	14604	38128	5390	5406	7117	3988	6228	5004	5979
**hsa-mir-1199-3p**	4406	6048	3910	8016	3891	3714	4099	5828	5285	4301	2994	5025	3435	2417	5498	4689	2851	4462	5620	1905	2655	4023
**hsa-mir-1199-5p**	4097	5629	2568	8743	4643	4302	2094	5890	3606	4234	1743	4432	4841	3272	1952	4509	3384	2494	6287	1923	4360	4760
**hsa-mir-1200**	3948	4669	4405	9519	6428	4192	4784	5854	5726	4869	4300	5126	4953	3641	3408	5067	4756	4937	8603	3379	2387	4595
**hsa-mir-1202**	3837	3348	4154	4799	5279	2561	4569	4223	4109	3403	4991	4270	3077	3762	1763	3187	5522	4972	3987	4098	1977	4478
**hsa-mir-1204**	3774	8042	18468	7285	14137	13374	26413	11514	24512	11552	21624	12165	7473	11840	21409	11699	6631	22530	6627	12606	4463	8764
**hsa-mir-1207-3p**	3542	7805	7809	3196	7459	5715	5785	8693	9751	6875	7877	6809	9735	6978	9848	12670	7968	13830	3415	8627	5349	9128
**hsa-mir-1207-5p**	3330	2369	1859	5402	2607	2198	1462	2546	3083	2633	1610	3093	2170	1694	1537	2945	2512	2136	2471	1527	2611	2562
**hsa-mir-122-3p**	3252	7572	5181	4267	6585	4640	4255	6553	7836	4610	10701	6108	5648	5529	13936	5074	7693	6605	4119	5068	4025	5773
**hsa-mir-1224-3p**	3224	2394	3120	1447	4165	2340	2914	1418	4287	3819	5475	4608	3747	2818	753	3280	5653	2646	2391	4288	6938	3117
**hsa-mir-1224-5p**	2774	3479	3072	2639	3262	3437	4641	1884	3100	2653	2543	2731	2308	2759	5344	3592	2323	2180	2785	3457	1949	3769
**hsa-mir-1225-5p**	2757	2598	2227	1351	2300	3577	3525	966	2593	2569	1701	1111	2225	4166	9351	1774	1846	1672	2307	2325	3148	2643
**hsa-mir-122-5p**	2726	1627	1132	4175	939	1848	888	3423	961	1925	746	1500	2698	1138	1266	1931	1759	737	4577	1169	730	2283
**hsa-mir-1226-3p**	2720	2110	1584	1277	2631	2845	1562	716	2096	3347	1259	1052	2150	7625	8769	1060	1340	953	1451	1815	5289	1549
**hsa-mir-1226-5p**	2703	3424	5314	5810	5349	7080	5648	7705	6015	6207	6232	4858	4918	5552	10973	6427	5461	6056	5826	5340	3808	4849
**hsa-mir-1227-3p**	2568	2816	3751	1330	2308	1914	3026	1433	2701	2069	3491	3042	1811	2099	1819	1939	2460	2782	1804	2534	1357	1639

**hsa-mir-1227-5p**	2471	1863	1439	3635	1605	1750	1118	1783	1315	1515	1176	1637	1278	1236	565	1184	2111	992	1019	1398	1107	1329
**hsa-mir-1228-3p**	2416	2787	2654	2330	2586	2529	2737	2607	2728	2905	3267	3154	3358	2477	2820	2874	2712	2075	3303	2770	2453	2441
**hsa-mir-1228-5p**	2408	2376	1785	1082	834	3257	1856	3346	1039	1529	1067	1055	1398	3369	1085	2764	1725	2383	2411	2528	5127	1584
**hsa-mir-1229-3p**	2259	1004	642	1954	806	649	656	1751	630	580	1386	713	232	1203	1138	419	1054	2060	573	631	460	921
**hsa-mir-1229-5p**	2024	1838	1907	1252	2048	1423	1832	709	2046	1556	2137	1982	1022	1064	548	1163	2173	1492	1581	1485	840	1116
**hsa-mir-1233-5p**	2018	2554	1756	5373	2756	1226	1189	2453	2548	2991	1278	3397	3871	981	2680	2173	1728	2357	3069	961	664	2768
**hsa-mir-1234-3p**	1947	2460	4494	1628	2075	2350	5765	2079	3077	1941	4063	2188	1260	1943	2253	1915	2202	4367	2171	3120	640	2163
**hsa-mir-1236-5p**	1930	3508	3489	5806	1691	2670	5568	2848	1512	2012	1602	2964	1760	2258	2160	1552	2435	2441	5944	2470	857	1886
**hsa-mir-1237-3p**	1927	1641	2036	1596	2268	1396	1419	661	1957	1696	1881	1639	1946	1330	306	2045	2079	1603	1669	2000	2893	1558
**hsa-mir-1237-5p**	1804	5691	4160	4731	3628	4367	6092	4579	3666	3424	2583	3713	3774	2565	3541	3540	2903	4157	5785	3434	1692	3220
**hsa-mir-1238-5p**	1692	1958	2378	2756	2828	3778	2277	2888	2477	2714	2072	2913	3145	3020	1581	2512	2274	2066	2750	2393	1765	2458
**hsa-mir-1243**	1678	222	160	223	99	891	134	13	61	473	51	21	69	1267	2337	36	24	83	119	252	932	61
**hsa-mir-124-3p**	1629	1517	3620	465	1368	1550	7608	2202	2163	1168	4870	600	1932	8543	42021	1030	1750	5347	1431	3933	3711	2258
**hsa-mir-1244**	1629	1605	869	1259	1736	2011	596	1319	1303	1451	1148	1451	3422	1292	319	1588	2525	851	1493	1693	5035	2027
**hsa-mir-1245a**	1612	1579	2179	2522	1577	2393	3800	1761	2179	1949	1676	1605	1194	2303	2293	2408	1192	3032	1631	1523	1357	1838
**hsa-mir-1245b-3p**	1592	309	434	319	123	12751	196	175	21	124	17	238	538	684	82	315	79	57	704	284	3389	230
**hsa-mir-1245b-5p**	1560	3693	3767	6327	3981	5679	5486	4433	4105	3856	2867	4478	3909	3275	3945	2905	2714	2973	4103	3226	3665	3155
**hsa-mir-124-5p**	1475	3089	1932	6076	2233	1790	2048	2828	1654	2688	1226	2443	4010	1249	1099	2075	1881	1417	5661	1615	1173	2457
**hsa-mir-1246**	1452	666	554	518	545	860	440	279	660	737	665	465	755	996	1526	425	461	441	502	478	2773	450

**hsa-mir-1247-3p**	1426	731	378	2089	512	543	158	492	344	963	85	398	517	1102	261	317	305	298	623	452	2317	578
**hsa-mir-1247-5p**	1423	4072	6270	4824	4030	4707	5738	3548	4897	4099	4811	3809	2429	2759	2946	3410	3238	4072	4879	4534	754	2477
**hsa-mir-1248**	1400	1292	1478	692	1491	1251	1084	800	1697	1830	1964	1548	1973	1416	684	1543	2792	1506	1362	1935	2282	1756
**hsa-mir-1249-3p**	1389	1335	1358	387	513	7604	1141	1065	677	857	337	767	2135	2906	8521	2206	393	841	1294	1259	3582	1877
**hsa-mir-1250-5p**	1386	1576	1386	3167	1548	3134	1275	2025	863	1360	406	1828	1951	1286	653	1526	967	844	1530	1436	1572	1519
**hsa-mir-1251-5p**	1326	2012	2124	1990	2214	1627	5476	1604	2622	1624	2295	1508	1224	1208	6494	2319	1157	2454	1937	1744	851	2070
**hsa-mir-1252-5p**	1283	855	756	1940	875	554	695	2073	418	637	182	615	753	463	1141	434	171	380	1518	461	147	899
**hsa-mir-1253**	1194	1637	1610	3040	1570	2305	2652	1611	1297	2013	1040	1903	1837	1328	956	1529	1550	1429	3559	1320	581	1828
**hsa-mir-1254**	1188	993	1160	567	1725	1292	789	1186	1879	1387	2267	1355	2101	1212	453	1661	2747	1504	1246	1876	2181	1731
**hsa-mir-1255a**	1134	2260	1349	1614	1937	1380	1437	1894	2022	1274	2463	1735	1515	1329	3462	1573	2073	1841	1970	1333	1245	1551
**hsa-mir-1255b-5p**	1128	2656	2025	1344	2056	3198	2350	2101	2593	1547	1659	2027	4110	2112	1250	2627	1924	3004	1755	2260	4509	1578
**hsa-mir-1256**	1122	2358	2234	794	1701	1705	2141	1672	2118	1347	1968	1672	1171	1607	2731	1426	1724	1639	1230	1619	636	1286
**hsa-mir-1257**	1091	1259	617	578	991	755	782	712	1177	596	1783	888	481	594	733	740	1342	1058	793	710	427	879
**hsa-mir-1258**	1048	91	71	145	59	47	43	69	32	36	44	71	78	275	33	120	200	244	17	394	116	440
**hsa-mir-125a-3p**	1016	866	1128	802	1015	790	1072	1138	1373	1264	1895	1002	1183	881	940	1203	1910	993	1043	1190	756	912
**hsa-mir-125a-5p**	942	1361	1610	1220	1140	1266	1244	1071	1875	1206	1980	1156	858	829	1132	1202	1231	1590	1368	1426	300	983
**hsa-mir-125b-1-3p**	936	331	222	316	904	257	311	233	291	193	1085	221	324	335	116	359	276	526	108	539	728	392
**hsa-mir-125b-2-3p**	931	648	432	1224	591	380	535	957	820	550	555	655	326	304	103	693	507	745	484	317	243	565
**hsa-mir-125b-5p**	882	269	337	298	345	323	458	479	252	224	276	166	232	275	250	173	171	167	201	148	191	287
**hsa-mir-1260a**	882	1328	2131	716	1429	1521	2009	1208	2610	1600	2460	1367	1787	1265	1861	2999	2681	1879	1371	2696	1214	3026

**hsa-mir-1260b**	868	1365	158	25	53	123	444	229	54	92	54	79	71	1299	4133	63	106	103	123	72	1451	108
**hsa-mir-1261**	859	633	599	1394	729	417	423	928	735	659	596	807	668	301	432	639	508	918	841	314	167	635
**hsa-mir-1262**	850	644	738	1912	680	1539	1425	1068	517	938	993	778	1078	489	908	583	454	888	2203	380	574	533
**hsa-mir-1263**	850	881	669	1341	810	5314	578	982	465	590	270	771	1276	1176	522	840	460	649	1673	680	1155	940
**hsa-mir-126-3p**	819	997	736	1298	1296	798	966	608	932	1459	1065	1837	672	1061	870	578	947	447	1680	744	682	681
**hsa-mir-126-5p**	819	175	139	326	35	150	456	73	52	115	419	264	323	601	161	138	41	496	577	229	364	499
**hsa-mir-1266-5p**	793	1084	1094	1224	1003	824	1684	1793	1175	882	1170	964	794	943	1925	1008	942	1930	1173	1090	456	1087
**hsa-mir-1267**	779	240	56	1819	117	213	24	667	42	185	19	171	255	86	23	246	179	197	259	148	151	210
**hsa-mir-1268a**	744	702	780	844	1055	687	725	611	1132	955	1412	1321	669	1035	511	789	1214	518	888	845	1434	824
**hsa-mir-1268b**	713	1208	1071	1479	1240	2473	1133	1586	1113	1528	1020	973	1732	2664	2468	1984	1040	1332	1022	1165	1497	1204
**hsa-mir-1269a**	693	200	332	603	1101	201	446	527	450	376	1760	408	285	912	271	632	773	604	169	1473	265	616
**hsa-mir-1269b**	690	408	230	277	349	356	216	196	364	340	363	332	406	480	368	379	408	257	386	365	1306	319
**hsa-mir-1270**	684	673	232	1589	219	1338	126	1132	63	375	84	415	678	251	67	391	246	73	498	177	1716	321
**hsa-mir-1271-5p**	676	451	388	919	450	658	270	617	467	545	248	565	820	420	373	682	513	365	797	393	1028	717
**hsa-mir-1272**	673	953	795	560	850	636	663	506	855	621	996	921	504	575	1336	568	763	584	513	474	373	619
**hsa-mir-1273a**	659	480	332	337	220	219	181	149	371	119	462	310	269	430	271	322	597	361	336	741	1550	438
**hsa-mir-1273c**	656	1099	1274	883	1231	1186	1350	931	1169	1565	1573	1189	739	2813	4192	1246	1376	1234	712	1457	1267	1258
**hsa-mir-1273d**	656	1175	1001	1202	1362	1692	1043	2015	1302	1175	1000	1225	2604	1597	1374	1550	1044	1378	677	1363	2238	1212
**hsa-mir-1273e**	636	1350	1186	812	1289	2310	1413	614	1467	1463	865	1019	1371	2721	1668	1309	572	1200	901	1131	1515	648
**hsa-mir-1273f**	578	761	2046	397	826	596	1318	664	1386	1181	1402	1249	585	585	674	1134	1113	1004	562	1214	644	570
**hsa-mir-1273g-3p**	524	473	415	340	306	618	429	219	321	667	281	360	385	672	1969	347	207	205	357	375	1032	302
**hsa-mir-1273g-5p**	515	444	547	447	359	671	656	213	430	525	369	434	196	535	695	325	284	492	300	375	294	214
**hsa-mir-1273h-3p**	512	808	797	475	657	753	726	448	566	667	602	693	596	536	828	531	552	481	760	505	732	519
**hsa-mir-1273h-5p**	501	473	172	348	206	213	147	140	171	220	359	166	122	353	142	135	210	439	104	118	395	116
**hsa-mir-127-3p**	487	1077	807	365	913	468	1342	746	783	501	1413	616	329	447	2289	461	663	961	988	772	307	611

**hsa-mir-1275**	484	95	179	89	130	933	174	90	100	499	85	64	127	1211	3142	177	125	86	144	228	951	165
**hsa-mir-127-5p**	481	589	661	1316	523	859	985	924	706	600	416	541	487	345	1463	613	428	864	822	353	239	468
**hsa-mir-1276**	472	1852	638	1167	1325	1110	456	1207	1152	715	428	780	2606	684	635	1255	904	592	1167	606	2113	962
**hsa-mir-1277-3p**	444	131	228	408	176	191	168	387	270	308	380	248	285	198	271	376	272	235	252	324	114	320
**hsa-mir-1277-5p**	441	58	77	326	70	144	39	139	53	232	55	80	221	141	88	275	160	63	238	126	395	219
**hsa-mir-1278**	435	1747	702	1436	1229	413	1238	1331	687	2371	796	1278	504	281	732	988	375	889	2561	915	26	1401
**hsa-mir-1281**	432	440	284	1717	322	737	487	611	279	380	302	443	471	219	371	302	286	678	728	251	289	308
**hsa-mir-128-1-5p**	429	811	533	1043	616	338	356	463	321	584	230	711	594	433	226	441	535	276	667	517	228	408
**hsa-mir-1282**	427	131	68	213	41	613	81	9	19	214	16	12	8	510	429	13	14	14	71	106	897	39
**hsa-mir-128-2-5p**	427	1194	1502	908	1188	1031	1565	1023	1644	1069	1500	1556	1012	911	859	1039	1229	1074	1097	1248	517	1128
**hsa-mir-1283**	412	408	419	497	483	352	360	486	566	408	836	479	457	545	875	440	667	521	183	522	796	502
**hsa-mir-128-3p**	407	502	369	277	602	150	380	140	383	324	660	535	293	282	294	296	572	321	381	395	241	275
**hsa-mir-1284**	398	346	426	262	288	466	450	282	425	447	272	485	269	181	173	326	325	238	247	336	403	217
**hsa-mir-1285-3p**	395	153	361	78	212	103	933	270	292	256	759	199	117	2605	2655	177	437	513	455	621	68	182
**hsa-mir-1285-5p**	389	611	542	802	430	303	462	657	642	439	419	481	347	341	1519	443	458	640	511	339	132	399
**hsa-mir-1287-3p**	387	1077	1212	943	947	1693	2145	713	987	927	625	910	813	665	607	932	499	1309	1285	838	500	674
**hsa-mir-1287-5p**	378	437	346	128	314	251	320	125	373	255	416	318	185	247	226	247	337	378	263	263	149	255
**hsa-mir-1288-3p**	358	84	108	82	40	251	303	18	44	124	55	21	81	479	2088	23	13	52	113	171	605	37
**hsa-mir-1289**	355	69	105	60	56	146	74	35	59	77	30	84	76	73	26	101	75	159	67	75	180	53
**hsa-mir-1290**	344	62	59	124	88	94	39	35	47	523	53	53	93	959	870	60	79	66	123	138	395	51
**hsa-mir-1291**	338	135	147	223	396	49	185	248	134	78	495	158	138	187	182	176	148	353	36	479	79	152
**hsa-mir-1292-3p**	329	215	318	53	179	281	165	391	246	227	310	182	414	282	109	713	442	745	114	512	1339	356

**hsa-mir-1292-5p**	312	182	120	287	79	207	130	163	106	136	73	136	76	110	119	168	171	184	222	107	83	95
**hsa-mir-1293**	306	127	263	167	310	116	725	251	251	276	539	162	168	2381	1456	130	392	383	582	706	164	167
**hsa-mir-1294**	301	25	39	28	8	150	18	36	20	24	6	22	28	55	75	59	36	43	28	48	300	51
**hsa-mir-1295a**	301	277	233	160	188	169	135	112	189	188	222	182	177	297	579	146	181	238	156	165	208	147
**hsa-mir-129-5p**	295	462	466	383	575	398	399	314	360	367	360	527	649	366	516	434	403	370	380	399	298	485
**hsa-mir-1296-3p**	295	619	492	855	418	457	443	677	251	533	264	619	549	429	408	505	602	304	1040	580	364	496
**hsa-mir-1296-5p**	295	684	975	518	573	867	1062	639	678	781	1132	1027	694	796	233	892	911	657	588	1333	224	441
**hsa-mir-1297**	286	175	158	504	148	313	281	425	113	188	87	204	340	103	75	321	201	180	156	276	237	279
**hsa-mir-1298-3p**	286	189	257	376	265	340	375	245	200	359	233	343	436	303	119	270	468	159	483	321	208	287
**hsa-mir-1298-5p**	283	36	71	82	54	12	100	163	186	64	132	111	57	103	88	76	136	90	55	124	26	94
**hsa-mir-1299**	266	131	143	248	199	74	117	206	118	159	184	169	199	189	82	184	212	160	165	213	105	228
**hsa-mir-1301-3p**	266	306	237	443	485	660	183	287	383	447	378	370	806	330	364	264	492	252	605	332	708	537
**hsa-mir-1301-5p**	258	757	611	621	1051	607	656	603	1011	505	1385	649	928	846	709	701	761	948	491	824	1138	667
**hsa-mir-1302**	258	135	59	85	208	40	62	67	185	294	1069	388	234	346	33	350	310	73	796	114	2210	195
**hsa-mir-1303**	252	579	311	202	542	841	330	136	207	1205	891	1018	395	789	159	172	762	521	1758	404	294	176
**hsa-mir-1304-3p**	243	98	114	85	236	219	158	88	233	245	98	99	185	357	418	124	116	103	119	140	226	189
**hsa-mir-1304-5p**	226	295	389	865	492	616	378	528	395	369	361	391	440	356	264	510	408	383	437	471	697	556
**hsa-mir-1305**	220	277	275	493	582	576	253	114	650	291	447	412	318	623	123	282	328	171	292	326	769	229
**hsa-mir-1306-3p**	215	360	602	798	300	489	502	506	256	659	190	649	696	412	270	992	359	364	517	418	384	683
**hsa-mir-1306-5p**	212	73	211	71	226	28	201	251	154	114	139	125	158	334	264	116	198	588	62	342	186	300

**hsa-mir-1307-3p**	209	62	133	50	80	71	411	67	111	112	203	69	77	660	212	60	136	151	249	185	243	39
**hsa-mir-1307-5p**	209	291	443	390	516	198	478	251	444	432	618	410	175	320	324	316	617	443	441	564	37	357
**hsa-mir-130a-3p**	200	204	413	312	160	533	552	600	266	457	222	361	323	137	67	376	223	214	428	429	180	356
**hsa-mir-130a-5p**	200	131	181	262	480	146	259	350	138	153	543	149	254	275	313	344	123	296	156	596	162	387
**hsa-mir-130b-3p**	189	65	72	57	47	107	48	44	62	55	74	69	68	88	137	78	73	92	38	86	199	32
**hsa-mir-1321**	189	386	319	348	609	296	280	282	399	281	503	391	365	326	170	253	409	281	229	372	250	273
**hsa-mir-1323**	186	171	161	273	192	283	165	178	172	197	140	186	137	205	1073	111	110	153	205	77	653	128
**hsa-mir-132-3p**	178	120	97	106	122	457	68	64	105	50	107	84	134	204	109	93	100	95	62	119	189	90
**hsa-mir-132-5p**	175	51	167	99	233	36	139	268	88	66	68	186	191	253	89	142	176	283	137	223	410	470
**hsa-mir-133a-3p**	175	262	80	2469	218	182	40	624	148	200	59	291	229	84	261	312	226	359	225	215	239	330
**hsa-mir-133a-5p**	172	84	101	234	102	329	167	235	129	81	99	134	184	162	215	172	147	210	253	125	224	197
**hsa-mir-133b**	172	589	644	294	309	515	608	397	513	380	408	355	444	296	473	478	313	499	375	402	189	351
**hsa-mir-1343-3p**	169	116	118	163	126	144	144	144	110	98	110	122	60	60	67	94	106	98	248	105	44	101
**hsa-mir-1343-5p**	166	84	85	131	92	75	105	86	73	99	106	119	100	71	81	140	146	130	76	81	39	137
**hsa-mir-134-5p**	163	193	137	124	166	211	128	80	207	150	187	164	199	189	275	191	168	126	116	146	246	195
**hsa-mir-135a-3p**	157	91	129	32	66	176	232	26	69	149	62	35	123	595	586	38	29	70	51	200	265	12
**hsa-mir-135a-5p**	157	29	59	60	26	36	117	31	61	90	81	47	8	201	369	40	49	138	98	66	72	59
**hsa-mir-135b-5p**	152	746	1062	287	408	851	1290	150	936	593	926	635	530	885	387	595	373	697	328	761	392	358
**hsa-mir-136-3p**	149	11	9	57	3	19	10	12	9	18	8	11	44	61	33	33	18	20	9	30	134	26
**hsa-mir-136-5p**	146	102	81	138	167	140	155	104	112	100	190	101	150	184	247	151	99	151	122	164	237	136

**hsa-mir-137**	143	218	362	177	218	109	307	451	270	179	238	186	206	156	75	303	183	372	170	253	167	325
**hsa-mir-138-1-3p**	143	109	93	323	194	168	95	165	149	127	187	151	220	131	61	167	253	100	145	123	270	224
**hsa-mir-138-2-3p**	140	29	133	67	99	38	357	71	109	145	192	84	48	686	187	69	105	113	62	208	50	25
**hsa-mir-138-5p**	137	273	196	184	155	409	247	115	171	238	106	174	171	285	859	160	136	139	358	139	66	120
**hsa-mir-139-3p**	135	18	38	149	28	35	33	139	31	15	29	34	13	52	37	120	27	39	72	35	85	52
**hsa-mir-139-5p**	129	95	309	216	159	191	254	356	258	268	314	160	310	166	445	339	288	255	154	386	373	404
**hsa-mir-1-3p**	129	76	46	199	105	133	40	50	42	88	40	82	188	109	33	96	188	64	155	65	156	131
**hsa-mir-140-3p**	129	189	336	220	150	235	299	401	379	396	159	283	325	111	263	371	392	267	174	421	85	261
**hsa-mir-140-5p**	123	106	187	298	224	155	183	162	283	204	347	310	129	263	116	177	330	118	234	216	313	196
**hsa-mir-141-5p**	120	87	60	46	99	146	41	130	128	49	30	73	167	149	373	177	62	80	24	57	318	179
**hsa-mir-142-5p**	115	73	72	184	138	157	140	130	75	128	55	121	159	60	18	124	95	64	154	73	53	107
**hsa-mir-143-3p**	115	36	63	11	41	22	43	63	23	22	21	45	54	77	186	24	47	111	36	50	64	74
**hsa-mir-143-5p**	115	47	68	35	38	69	51	22	36	48	48	66	102	46	18	25	59	41	108	43	4	51
**hsa-mir-144-3p**	112	175	217	14	83	254	238	35	165	189	121	104	125	226	161	114	86	114	84	185	110	90
**hsa-mir-144-5p**	109	29	50	89	32	94	88	25	68	55	32	31	26	58	35	42	22	50	88	37	64	30
**hsa-mir-145-3p**	109	55	71	43	97	48	69	155	35	41	31	47	139	106	32	55	49	166	31	100	39	93
**hsa-mir-1468-5p**	106	40	67	50	63	47	71	107	35	49	85	73	42	66	270	55	68	40	114	70	4	79
**hsa-mir-1469**	106	146	79	195	145	110	70	172	128	130	47	123	99	56	103	97	106	92	180	52	66	118
**hsa-mir-146a-3p**	103	80	419	35	124	61	617	80	118	116	113	129	249	434	299	133	181	329	144	445	224	224
**hsa-mir-146a-5p**	103	98	222	170	77	486	466	64	137	213	130	176	80	247	236	114	73	142	189	109	373	85
**hsa-mir-146b-3p**	103	215	222	39	56	439	843	500	944	116	56	135	794	451	1118	3283	91	175	170	354	333	1388
**hsa-mir-1470**	100	15	125	57	111	43	192	155	64	81	48	88	76	234	107	47	49	324	192	187	20	122
**hsa-mir-1471**	100	4	57	21	26	62	44	28	60	53	31	67	33	18	44	46	67	28	10	45	4	20
**hsa-mir-148a-3p**	100	91	81	39	90	45	67	44	79	70	88	70	51	98	96	31	90	49	113	68	156	68

**hsa-mir-148a-5p**	100	18	54	142	122	17	63	48	41	62	32	29	92	135	39	40	48	61	24	50	294	51
**hsa-mir-148b-3p**	97	7	10	4	26	53	29	6	22	13	20	18	21	32	14	20	7	7	8	28	103	31
**hsa-mir-150-5p**	94	91	4	50	23	70	7	53	3	10	1	9	65	24	124	16	14	12	126	2	219	19
**hsa-mir-151a-3p**	92	33	37	43	46	19	46	13	36	46	32	36	59	65	187	53	59	36	70	29	147	30
**hsa-mir-151a-5p**	92	76	169	32	99	53	223	107	165	50	394	60	129	710	1227	107	152	163	56	222	289	152
**hsa-mir-151b**	92	116	121	92	85	87	122	55	82	160	138	122	24	83	103	119	92	105	1	118	184	108
**hsa-mir-152-3p**	92	73	91	145	102	38	53	114	69	61	50	121	97	86	37	90	91	61	85	85	55	89
**hsa-mir-152-5p**	89	44	92	18	52	17	67	63	41	44	29	53	69	128	75	44	56	159	44	79	35	119
**hsa-mir-153-3p**	89	73	79	32	98	89	105	86	102	85	111	97	66	75	109	142	93	88	66	71	151	109
**hsa-mir-153-5p**	89	200	100	128	83	122	72	118	63	138	67	105	122	99	166	113	92	81	88	102	79	91
**hsa-mir-154-3p**	83	84	40	223	33	70	15	74	17	57	6	37	102	42	25	26	59	17	69	31	162	40
**hsa-mir-154-5p**	83	40	52	28	54	21	78	60	56	46	63	41	47	27	61	63	45	47	108	54	42	34
**hsa-mir-155-5p**	80	58	112	89	143	84	168	165	117	110	150	92	87	64	98	72	111	163	108	77	105	102
**hsa-mir-15b-3p**	77	226	187	351	288	147	156	207	212	181	336	252	218	145	284	169	297	174	202	197	195	244
**hsa-mir-15b-5p**	77	33	83	71	151	28	83	108	143	74	183	129	62	161	144	47	21	23	95	53	37	91
**hsa-mir-16-1-3p**	77	164	216	82	142	125	234	107	329	177	234	186	242	128	194	188	130	251	22	249	197	156
**hsa-mir-181a-3p**	72	55	61	14	35	40	52	34	110	43	71	89	53	84	14	84	61	58	34	47	235	46
**hsa-mir-181a-5p**	72	62	31	32	29	21	37	47	38	44	38	60	36	20	16	17	60	28	177	13	59	30
**hsa-mir-181b-2-3p**	72	80	161	32	91	89	165	79	119	184	94	210	78	46	61	86	95	97	140	98	217	76
**hsa-mir-181b-5p**	69	95	141	89	119	83	145	136	121	86	181	110	163	72	203	67	116	117	125	104	28	110
**hsa-mir-181c-3p**	69	47	62	96	59	113	56	160	133	61	85	63	138	53	32	216	143	148	10	117	178	134

**hsa-mir-181c-5p**	69	33	11	14	18	17	7	10	3	12	1	13	15	28	2	6	9	7	4	9	114	10
**hsa-mir-181d-3p**	69	65	30	32	36	34	41	66	36	30	11	32	16	23	33	39	9	19	28	19	33	27
**hsa-mir-1825**	69	207	117	99	92	202	104	149	110	118	120	105	126	156	210	131	113	122	252	83	90	102
**hsa-mir-182-5p**	69	58	19	28	21	71	15	23	20	31	41	22	56	27	58	25	53	14	90	16	114	49
**hsa-mir-183-5p**	66	73	75	60	73	62	99	76	93	61	51	71	78	41	96	72	50	111	66	53	46	69
**hsa-mir-185-3p**	66	226	120	259	90	519	187	158	88	221	54	54	110	616	1316	136	76	112	125	145	925	143
**hsa-mir-187-5p**	60	69	191	85	124	32	175	98	92	73	58	58	336	287	226	91	158	159	201	258	458	217
**hsa-mir-188-3p**	60	29	33	167	50	38	36	130	48	77	21	79	49	30	23	71	31	34	34	41	22	92
**hsa-mir-18b-3p**	57	15	33	103	53	98	92	38	53	37	64	66	39	52	44	81	69	103	70	67	85	90
**hsa-mir-1908-5p**	54	131	85	4	80	63	45	35	55	304	270	216	46	222	123	43	308	283	267	105	68	72
**hsa-mir-1909-5p**	54	47	159	64	125	159	201	140	136	93	125	90	87	114	74	94	101	204	169	98	33	81
**hsa-mir-190a-3p**	54	33	155	131	115	145	160	194	118	174	118	212	162	102	65	180	107	106	80	127	46	137
**hsa-mir-1912**	52	80	231	82	140	28	270	31	97	60	117	60	249	285	236	59	114	271	76	232	199	106
**hsa-mir-1914-5p**	49	36	65	50	45	70	70	88	41	54	70	45	51	56	96	63	78	71	53	57	28	78
**hsa-mir-192-3p**	49	29	68	32	67	189	75	206	48	43	23	44	303	63	116	203	87	127	51	137	270	184
**hsa-mir-193a-5p**	46	58	64	57	86	111	91	76	80	43	66	92	61	50	18	49	86	93	132	70	107	83
**hsa-mir-194-3p**	46	116	63	78	43	110	37	53	30	82	12	35	30	149	105	69	26	39	44	52	59	71
**hsa-mir-195-5p**	43	138	149	170	128	202	141	197	115	130	146	163	146	197	138	167	117	121	126	123	140	175
**hsa-mir-196a-5p**	43	22	27	7	22	12	24	10	40	46	35	62	27	11	23	37	49	31	14	37	149	16
**hsa-mir-199a-5p**	43	15	51	25	37	27	59	41	49	39	16	34	56	149	54	37	37	136	37	120	33	123
**hsa-mir-19b-3p**	40	29	27	25	26	36	41	7	41	26	27	39	76	38	14	56	34	27	95	30	96	22
**hsa-mir-200b-3p**	37	142	299	206	148	157	266	238	248	131	273	141	94	125	224	274	156	285	156	310	42	150

**hsa-mir-203b-3p**	37	65	38	174	99	263	41	28	29	45	66	87	48	41	74	55	136	38	83	80	107	79
**hsa-mir-208a-3p**	34	44	262	53	72	101	408	16	49	90	25	60	129	200	152	94	86	119	79	146	500	110
**hsa-mir-211-5p**	34	29	24	4	11	16	16	7	12	7	2	13	11	29	16	35	10	11	17	17	18	12
**hsa-mir-212-5p**	31	51	71	35	94	17	103	66	71	68	108	101	36	33	79	60	76	78	81	108	22	28
**hsa-mir-221-3p**	29	106	36	28	24	58	39	117	45	38	14	41	93	24	51	47	24	26	81	30	140	73
**hsa-mir-223-5p**	29	15	11	21	29	41	23	13	22	7	18	23	34	44	18	15	19	16	22	21	123	20
**hsa-mir-22-3p**	29	25	16	14	23	17	4	3	13	2	20	11	6	54	23	22	9	25	1	21	31	8
**hsa-mir-224-5p**	29	291	496	135	299	265	534	321	465	358	352	283	241	268	266	268	325	452	332	373	20	277
**hsa-mir-25-3p**	23	25	57	21	29	32	59	79	42	29	118	29	28	129	184	42	83	39	55	59	191	50
**hsa-mir-2681-3p**	23	22	48	21	39	60	38	1	37	53	38	56	10	55	63	49	40	28	60	48	42	47
**hsa-mir-2681-5p**	23	84	22	60	50	25	48	36	37	37	103	40	100	127	103	123	42	90	60	65	103	174
**hsa-mir-26a-5p**	23	15	7	11	12	49	6	9	3	74	1	3	5	145	116	6	4	1	78	24	232	5
**hsa-mir-3074-3p**	17	25	19	60	15	66	10	117	12	27	10	24	62	26	28	48	41	23	11	29	11	42
**hsa-mir-3126-3p**	17	95	57	57	58	57	31	26	47	51	58	34	38	68	61	28	51	24	58	31	48	44
**hsa-mir-3126-5p**	17	116	29	231	53	71	25	104	49	56	17	75	75	5	23	46	20	47	119	24	9	54
**hsa-mir-3135b**	14	18	45	32	22	21	43	19	50	28	52	37	42	26	68	29	27	52	17	35	7	36
**hsa-mir-3138**	14	44	64	174	102	45	55	112	122	73	76	84	64	26	173	53	59	148	151	63	92	111
**hsa-mir-3158-5p**	11	7	18	43	16	10	14	35	10	26	12	31	16	13	4	14	26	13	10	11	33	5
**hsa-mir-3173-3p**	9	15	15	11	26	14	23	15	30	34	38	32	17	29	7	20	14	11	23	21	15	15
**hsa-mir-3195**	3	15	2	11	3	9	4	12	2	11	1	10	7	10	12	9	6	5	6	6	77	3
**hsa-mir-329-5p**	3	113	152	124	106	101	276	178	114	135	127	123	136	97	138	129	133	132	164	175	35	112

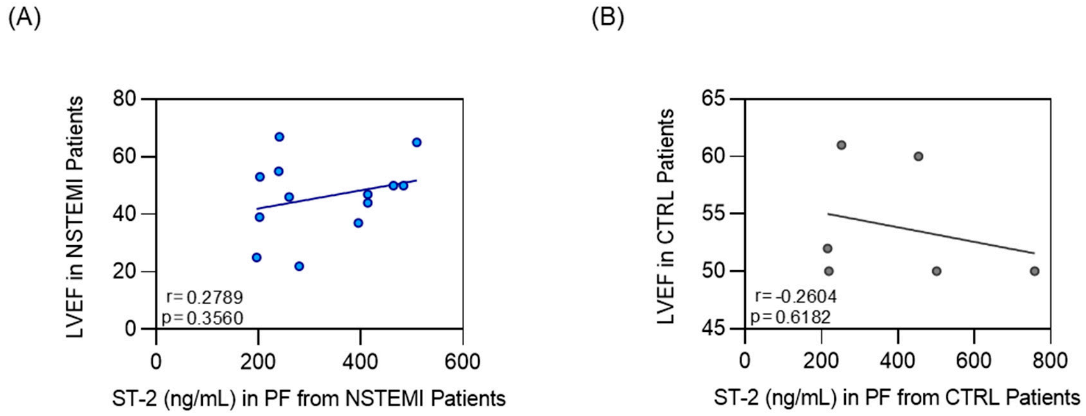

Supplementary Figure S1:

Spearman correlation between Left ventricle ejection fraction (LVEF) and ST-2 levels in PF from (A) NSTEMI (n=14) and (B) CTRL patients (n=6).

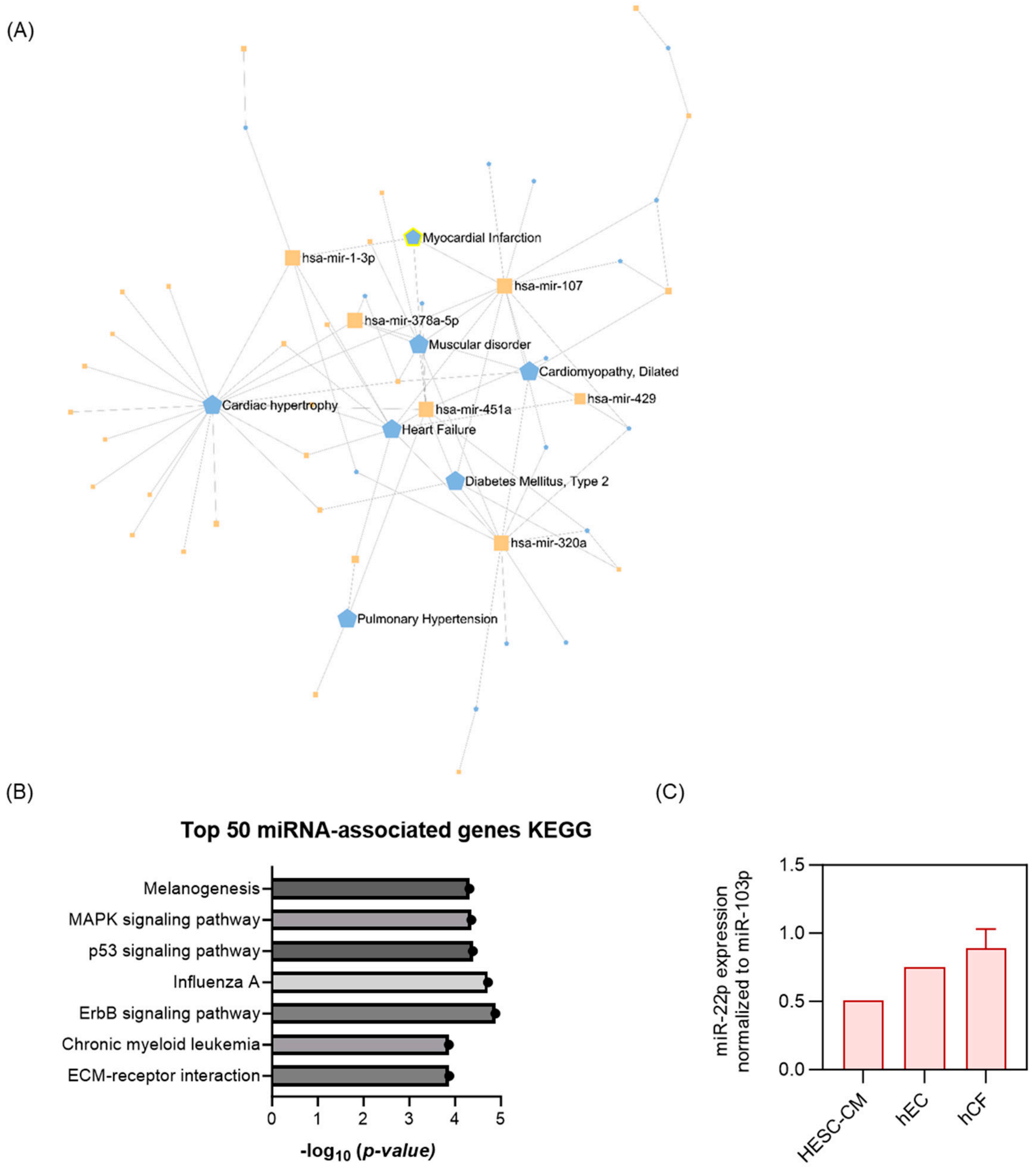

Supplementary Figure S2:

(A) Network analysis of disease-mIRs interactions using the the miRNet 2.0 online platform highlighting cardiovascular diseases entries. (B) Enrichment analysis of KEGG signaling pathways (miRNet 2.0) miRNA-target genes of the 50 most abundant miRNA in the PF. (C) miR-22-3p expression in human embryonic pluripotent stem cells (ESC)-derived ventricular cardiomyocytes (HESC-CM) primary human cardiac microvascular endothelial cells (hEC) and primary human cardiac fibroblasts (hCF) (n≥1/group).
